# Laughing Horses

**Published:** 1882-04

**Authors:** 


### LAUGHING HORSES.

Some old-fashioned philosophers tell us that horses cannot laugh, but I never believed it. If any of our boys and girls would like to see their good old family horse laugh, I can tell you how to bring the smiles to his face.

When your mother gives you a piece of cake or pie that you did not expect, I have seen the smiles creep over your faces, and if nuts were added you would laugh outright.

Do the same by your horse. He gets tired of dry hay, and enjoys an apple or a bunch of fresh grass, or a piece of bread or cabbage leaves, just as you enjoy your nuts and pie.

Think how many nice rides he has given you and how much hard work he has done, without a word of praise or an extra dinner. Give him a nice bit now and then, and see if he doesn't laugh and watch for your coming, as you watch for your father's when he comes from town about holiday time.

---

Use of Lemons.—For all people, in sickness or in health, lemonade is a safe drink. It corrects biliousness ; it is a specific against worms and skin complaints. The pippins, crushed, may also be mixed with water and sugar and used as a drink. Lemon-juice is the best anti-scorbutic remedy known. It not only cures the disease, but prevents it. Sailors make a daily use of it for this purpose. A physician suggests rubbing of the gums daily with lemon-juice to keep them in health. The hands and nails are also kept clean, white, soft and supple by daily use of lemon instead of soap. It also prevents chilblains. Lemon used in intermittent fever is mixed with strong, hot black tea or coffee without sugar. Neuralgia may be cured by rubbing the part affected with a lemon. It is valuable also to cure warts and to destroy dandruff on the head, by rubbing the roots of the hair with it. In fact, its uses are manifold, and the more we apply it externally the better we shall find ourselves.

### LIGHT.

DARKNESS, both in the mental and the physical world, is rapidly disappearing in our day. We say rapidly, in comparison with the progress made in former ages. Not only has the general diffusion of knowledge dispelled the darkness of ignorance and enlightened the minds of men as never before, but the darkness of night which has so long limited the activities of man is fast disappearing before the inventions of the day. In the early ages, Nature, as DeQuincey says, was too poor to afford her children candles. Mankind toddled off to bed at sundown. The ancients were forced to give all their amusements by daylight through lack of artificial light.

In our day we have seen the means of lighting the world advanced from tallow candles to electricity. What a wonderful leap? To realize the extent of this advance, one must go back to the days of candle-light and advance step by step through whale oil, camphene, burning fluid, kerosene and gas, and then take a long leap to the pure light of electricity.

This abolition of darkness has not been accomplished without great labor and cost. Boston pays nearly half a million yearly for its street lights. Gas was a wonderful advance on the oil lamps, and made the world better worth living in. How much it has done to lessen crime in city streets we have only to go back to the scenes enacted in dark streets of old-time cities to fully realize.

But now comes a light in comparison with which the rays of gas are yellow, jaundiced and feeble indeed. Electricity, with its clear white flame, gives us the next thing to sunlight. It takes possession of the night while the sun goes about its work on the other side of the world. We can well spare the great luminary when it leaves behind so acceptable a substitute. What possibilities it opens up! Light, heat and motive power from one inexhaustible source! All limitation of business and industry disappears. It becomes practicable to work as well by night as day. Factory operatives, miners, clerks, can pursue their labors free from the noxious heat and vapors generated by gas. All evils which love the darkness will flee before this pure light. It will give greater safety to ships at sea, and illumine even the fogs which shroud great cities.

But the new light will not be without its dangers at first, which we must learn to avoid. Perhaps not the least of these will be the facility of turning night into day. How will busy man learn to stop when no night comes to bid him rest? This thing is another temptation to over-work which must be resisted. Then there is the danger from the electric lighting wires, which may turn incendiaries under unskillful management. But gas has greater dangers even than those of electricity, and we shall learn to guard against the one as we have the other. One thing is clear, electricity has come to stay. We must make the best of it, and that it will prove a great good in many ways there can be no doubt.

